# 

**Published:** 1894-08-18

**Authors:** 


**Notices of Births, Deaths, and Marriages** are inserted in this column at a charge of 2s. 6d. for an announcement not exceeding four lines.

**Notice to Advertisers and Subscribers.**

All Advertisements, Orders for Copies of The Hospital, and Business Communications should be addressed to **The Publisher, NOT TO THE EDITOR,** The Hospital, 428, Strand, W.C.

The Cost of Subscription, post free, is as follows:—Per week, 2½d.; per quarter, 2s. 8d.; per half-year, 5s. 3d.; per annum, 10s. 6d. Foreign:—Per Annum, 12s. 8d.; payable, in all cases, in advance.

### NOTICE TO CORRESPONDENTS.

All MS., Letters, Books for Review, and other matters intended for the Editor should be addressed The Editor, The Lodge, Porchester Square, London, W.

The Editor cannot undertake to return rejected MS., even when accompanied by stamped directed envelope.

**"Burdett's Hospital Annual," 1894.**

Fifth year of publication. 900 pp. Boards, gilt lettering. A most complete guide to British, Colonial, Indian, and American Hospitals, Dispensaries, Nursing and Convalescent Institutions, and Asylums. Price 5s. Published by The Scientific Press (Limited), 428, Strand, W.C.

**NOTICE.—The Index for Vol. XV. (ending March 31st, 1894) is on sale at the Office of this Journal, price 2½d., post free.**

### THE STUDENT OF MEDICINE.

### APPOINTMENTS.

T. W. Butcher, M.B., C.M.Edin., appointed Surgeon to the Blackpool Hospital. F. A. Elkins, M.B., C.M.Edin., appointed Medical Superintendent to the Sunderland Borough Asylum. F. A. Heslop, L.R.C.P., L.R.C.S.Edin., appointed Surgeon to the Blackpool Hospital. Mr. C. H. Hill, appointed Medical Officer of the Box District of the Chippenham Union. G. W. Hill, B.Sc.Lond., M.D.Lond., appointed Surgeon in Charge of Aural Out-patients at St. Mary's Hospital. G. Home, M.B., C.M.Edin., appointed Assistant House Surgeon to the Northern Hospital, Liverpool. S. J. Hutchinson, M.R.C.S.Eng., L.D.S., appointed Consulting Dental Surgeon to the University College Hospital. G. C. Kingsbury, M.D., B.Ch.Dub., appointed Consulting Physician to the Blackpool Hospital. W. R. C. Middleton, M.A., M.B., C.M., D.P.H.Aberd., appointed Medical Officer of Health for Singapore Straits Settlements. W. J. Molloy, M.B., B.Ch., appointed Surgeon to the Blackpool Hospital. A. A. Mussen, B.A., M.B., B.Ch.Dub., appointed House Physician to the Northern Hospital, Liverpool. L. V. Parry, L.R.C.P., L.R.C.S., appointed House Surgeon to the Flintshire Dispensary. W. R. Richardson, M.R.C.S.Eng., appointed Surgeon to the Blackpool Hospital. J. Ruxton, M.D., C.M.Aberd., appointed Consulting Surgeon to the Blackpool Hospital. J. P. Simpson, M.D., C.M.Glasg., appointed by the London County Council Medical Officer to the Metropolitan Fire Brigade, No. 9 District. W. Whitelaw, M.D.Aberd., appointed Medical Officer for London County Council District, No. 10.

### VACANCIES.

### Resident Medical Officers.

Cumberland Infirmary, Carlisle.—House Surgeon. Salary £70 per annum, with board, lodging, and washing. Applications and testimonials to the Secretary before August 29th.

General Hospital, Nottingham.—Senior Resident Medical Officer; doubly qualified. Salary £120 for the first year, with an addition of £10 a year up to £150, with board, residence, and washing. Applications to the Secretary by September 1st.

Hants County Asylum.—Third Assistant Medical Officer. Salary £100 per annum, with furnished apartments, board, washing, and attendance. Applications and testimonials before August 23rd to Committee of Visitors, Knowle, Fareham.

Liverpool Infirmary for Children.—Assistant House Surgeon. Board and lodging, but no salary. Applications and testimonials to C. W. Carver, Hon. Secretary, by August 18th.

West Riding Asylum, Wakefield.—Fourth Assistant Medical Officer. Salary £100 per annum, rising £10 annually to a maximum of £150, with furnished apartments, board, washing, and attendance. Applications to the Medical Director by August 18th.

### Non-Resident Medical Officers.

Aberdeen Royal Infirmary.—Ophthalmic Surgeon. Applications by August 31st.

Burgh of Leith.—Medical Officer of Health. Salary to commence at £350. Applications and testimonials to T. B. Laing, Town Clerk's Office, Leith, by September 1st.

Cambridge University.—John Lucas Walker Studentship. Applications to Professor Roy, New Museums, Cambridge, by September 30th.

General Hospital, Birmingham.—Assistant-Surgeon. Appointment for three years, with eligibility for re-election. Honorarium, £100 per annum. Applications to Howard J. Collins, House-Governor, by September 1st.

### UNIVERSITIES AND COLLEGES.

### University of Edinburgh.

The following additional candidates have passed the Second Professional Examination for the Degrees of M.B. and C.M.: E. J. Bray, J. Eason, S. J. Gilfillan, M.A., J. B. Hay, B. L. L. Learmouth, S. M'Donald, J. D. G. M'Pherson, R. L. Moorhead, W. Mowatt, R. Owen, A. J. Park, S. H. Richards, D. Roger, A. H. Rutherford, T. D. Sadler, F. O. de Souza, G. E. Stewart, A. Wallace, H. J. Cardale, J. M. Coates, G. E. Elliott, R. M. Hall, H. Halton, P. T. Herring (with distinction), A. Y. Greenwood, L. Ker, G. F. Lundie, M.A., P. C. Lüttig, A. Macdonald, F. B. Macdonald, J. A. Milroy, M.A., F. J. R. Momple, W. A. W. Moore, H. M. Morton, O. St. J. Moses, W. A. Potts, B.A., R. J. Ralston, G. L. Roberts, F. M. Simmonds, J. A. Thwaits, J. B. Tough. The following candidates have passed in the first division of the same examination: P. W. Shephert, J. W. Simpson, T. H. Stevenson, A. E. Williams.

The following candidates have passed the First Professional Examination: A. L. Anderson, W. Bennett, G. Liddell, W. E. Roberts, E. M. Carlyle, S. Faed, H. C. Gibson, A. D. Grange, H. Malins, O. Raith, R. H. White-Jones, J. B. Wilson, C. J. Wotherspoon, S. C. Rose, R. W. T. Ewart, C. L. Maude, H. H. Roberts, J. Hunter, E. M. Lithgow, G. Thomson, A. E. B. Wood, P. H. Henderson, W. Lockerbie, P. E. Millard, A. B. Shed, W. N. Barker, J. M. Bowie, J. M. Campbell, G. Dick, C. King, G. E. T. King, R. King, P. L. M'All, J. W. Mackenzie, T. R. Matthews, *W. P. Meldrum, G. A. Rosie, I. Shalaby, J. A. C. Smith, W. Smith, H. Wade, D. Wardrop, D. B. Waters, W. J. Baird, C. M. Pearson, G. Lyon, H. Macfarlane, A. C. M'Gilchrist, T. B. H. Scott, R. Ashbury

### THE STUDENT OF MEDICINE—*continued.*

D. D. Farquharson, A. G. Hamilton, E. C. Peake, I. Aird, J. Alcindor, T. C. Caldwell, C. H. Durrant, C. H. G. Gard, F. J. Gray, A. A. Grinn, G. de Laba, D. A. MacVean, H. J. Patchett, A. de St. L. F. Perigal, *J. H. Rhodes, *A. C. Sandstein, L. J. M. Deas, R. Ritchie, C. H. G. Ritter, G. Fournie, E. R. Grey, A. E. Brookes, A. D. Fordyce, T. E. Hincks, J. M'Gibbon, A. S. Parker, G. B. Pemberton, C. M. Robertson, G. N. W. Thomas, C. S. Brebner, J. M. Cuthbert, J. Munroe, T. A. Price, W. L. Pritchard, G. W. Smith, R. C. Verley, A. S. Allum, T. R. W. Armour, W. Craig, C. H. Elmes, O. A. Gee, R. Gibson, A. Gilmour, A. C. Renwick, T. Henderson, M. Johnston. The asterisk denotes distinction.

Botany, Zoology, and Physics.—T. J. T. M'Hattie, R. M. Dalziel, J. Masson, M.A.

Botany and Chemistry.—A. M. Joy, J. Downey, J. A. Thomson, J. M. Ross, C. S. Stevenson.

Chemistry, Zoology, and Physics.—C. W. Donald.

Botany.—T. F. Blake, F. H. Dommise, E. G. Ffrench, P. A. Leighton, F. N. Menzies, F. A. Georgeson, W. J. M. M'Farlane, J. G. Bell, P. J. Bodington, G. J. R. Carruthers, E. W. Martin, R. Bruce, F. Burns, R. Cameron, R. G. Carr, M. Carthew, C. S. Clark, A. Dods, J. P. Douglas, C. Forsyth, A. W. Fuller, W. A. Gilbert, T. E. Gilbert, G. Hamilton, W. D. S. Harrison, H. T. Holland, C. A. B. Horford, A. Hunter, B. S. Hyslop, J. Jeffery, S. H. Johnston, A. C. Kirkpatrick, P. H. Macdonald, J. C. M'Conachy, E. M'Culloch, J. M'Gregor, D. N. M'Lagan, R. A. M'Neill, E. C. G. Maddock, T. Meldrum, G. H. Menzies, M.A.; J. R. Munro, F. Myers, H. X. Paxton, W. Raine, C. F. T. Scott, A. Shearer, C. E. Smith, F. H. Stirling, F. T. Thomson, H. E. Wareham, A. C. Brown, A. D. Cameron, J. F. G. Martin, F. P. Bester, E. F. Cyriax, G. E. V. K. Davidson, W. W. Garthwaik, P. Pattison, W. Purves, R. Rutherford, P. G. E. M. Toit, J. W. N. Thomson, A. Tweedie.

Botany and Zoology.—L. Kingsford, A. Macleod, R. M. Skinner, F. G. Ralston, G. H. Boyden, A. G Boyden, L. D. H. Baugh, G. H. J. Brown, J. C. Carr, W. Darling, A. H. Griffith, G. G. Hay, W. E. Hutchison, D. B. King, M.A., T. Livingstone, J. M. M'Donald, R. C. M'Lachlan, L. C. M'L. Wedderburn, F. C. Matthew, J. G. Munro, A. C. Murray, C. F. Noble, J. Orr, W. Park, M.A., L. J. L. de Pavillet, J. Pender, W. Rogers, J. A. Smail, A. J. Spiganovicz, W. J. Stuart, C. H. Watson, A. Whittome.

Zoology.—J. W. Falconer, A. Whyte, E. R. Branch, J. Husband.

Chemistry.—E. R. S. Hale, B. B. Head, J. Vare, J. M. Benson, G. O. Rowley, T. R. Robertson, J. S. Bostock, C. E. Hutson, F. G. Middleton, J. P. Thorne.

Physics.—W. W. Thom, H. H. Broome, H. G. P. Raeburn, J. R. Robinson, J. E. Ratcliffe.

Botany, Zoology, and Chemistry.—A. M. M'Intosh, H. W. Borcham.

Botany and Physics.—J. Cullen, W. J. Jones, D. M. Walker, P. S. Hopkins, F. E. Robinson, W. E. Tellet, A. H. Thomas, H. W. Young, E. A. Brown, D. A. Callender, H. G. Carlisle, H. L. Clift, B. J. Courtney, J. Craig, Y. A. Djidjizian, H. Faulkner, D. Ferrier, J. J. Galbraith, T. B. Gornall, G. Haddow, D. J. Hughes, H. C. Kerm, K. W. Konzinopoulos, G. D. Laing, G. R. Laing, J. Luckhoff, R. N. M'Donald, W. H. Mackay, M. Mackelvie, G. C. M'Leavy, C. W. F. Melville, M. C. Morgan, A. Mouat, T. P. Oates, D. Robertson, G. B. Robinson, H. D. Shepherd, A. B. Slater, A. K. Smith-Shand, N. D. Walker, H. D. Wilson.

Chemistry, Botany, and Physics.—A. R. Buchanan, L. Crossley, G. A. Mackay.

